# Building a successful international research community through data sharing: The case of the Wheat Information System (WheatIS)

**DOI:** 10.12688/f1000research.23525.1

**Published:** 2020-06-05

**Authors:** Taner Z. Sen, Mario Caccamo, David Edwards, Hadi Quesneville

**Affiliations:** 1 Western Regional Research Center, Crop Improvement and Genetics Research Unit, United States Department of Agriculture-Agricultural Research Service, Albany, CA, USA; 2 NIAB, 93 Lawrence Weaver Road, Cambridge, CB3 0LE, UK; 3 School of Biological Sciences and Institute of Agriculture, University of Western Australia, Perth, WA, Australia; 4 Université Paris-Saclay, INRAE, URGI, Versailles, 78026, France; 5 Université Paris-Saclay, INRAE, BioinfOmics, Plant bioinformatics facility, Versailles, 78026, France

**Keywords:** WheatIS, community, data sharing, bioinformatics, wheat

## Abstract

The International Wheat Information System (WheatIS) Expert Working Group (EWG) was initiated in 2012 under the Wheat Initiative with a broad range of contributing organizations. The mission of the WheatIS EWG was to create an informational infrastructure, establish data standards, and build a single portal that allows search, retrieval, and display of globally distributed wheat data sets that are indexed in standard data formats at servers around the world. The web portal at WheatIS.org was released publicly in 2015, and by 2020, it expanded to 8 geographically-distributed nodes and around 20 organizations under its umbrella.

In this paper, we present our experience, the challenges we faced, and the answer we brought for establishing an international research community to build an informational infrastructure. Our hope is that our experience with building wheatis.org will guide current and future research communities to facilitate institutional and international challenges to create global tools and resources to help their respective scientific communities.

## Introduction

In 2011, the ministers of agriculture from the G20 nations launched the Wheat Initiative in order to create an international umbrella organization to guide research priorities for developed and developing nations and facilitate communication between international organizations working on wheat ( www.wheatinitiative.org/). Under the Wheat Initiative, several “Expert Working Groups“ (EWGs) were formed to fulfill this mission. At the time of writing in 2020, there are 11 EWGs. Realizing the importance of findability and accessibility of wheat data sets distributed around the world, the Wheat Information System (WheatIS) Expert Working Group was established in 2012 to develop data standards for the wheat community and enable data query and access to globally-distributed data sets in standardized formats. The core collaborating groups was chaired by members from The Genome Analysis Centre (TGAC, now Earlham Institute) in the United Kingdom, l’Unité de Recherche Génomique Info (URGI) in France, the United State Department of Agriculture, Agricultural Research Service (USDA-ARS) in the US, and University of Queensland in Australia.

Such a multi-faceted global data indexing and sharing challenge required close, sustained, and dedicated collaboration among wheat researchers with overlapping expertise. Three years after the inception of the WheatIS EWG, the WheatIS portal was made publicly available in 2015 through wheatis.org. The computational infrastructure, web presence, and data content were created by the EWG committee members, other scientists, programmers, and technicians. Currently, the web portal is maintained at the University of Western Australia, Australia, and the portal WheatIS servers are located at the “Plant Bioinformatics Facility” hosted by URGI France. Only after 7 years, WheatIS “nodes,” i.e., servers that contain indexed and formatted data sets, proliferated and are currently distributed in 3 continents in 5 countries, demonstrating the buy-in from the wheat research communities ( Alaux *et al.*, 2018; Blake *et al.*, 2019; Scheben *et al.*, 2019; Wilkinson *et al.*, 2016b; Yuan *et al.*, 2017).

In this paper, we describe our experience at forming our research community and building wheatis.org in order to provide our answers to the problem of executing such a large-scale project across borders, organizations, and funding mechanisms, so that other research communities can benefit from our experience. We nevertheless wish to mention that there is no single path to create such a global infrastructure and community, and our experience is only an example of how such a productive and successful collaboration can be built.

### The WheatIS Expert Working Group (EWG)

*Goals of WheatIS Expert Working Group*. The Wheat Initiative tasked the WheatIS EWG’s to provide the international wheat research community with easy access to wheat genetics, phenotype with environmental information, genomic data and bioinformatics tools, and to support and promote the diverse wheat databases internationally. Specifically, its goals are: 1) provide the wheat research community with a single-entry point of access to genetic, phenotypic, and genomic resources; 2) promote the development of services on top of existing wheat / Triticeae databases; 3) define guidelines for data curation, nomenclature, standards, and integration; and 4) provide a registry of wheat data resources.

*Building an expert working group*. The initial group was formed with a focus on recruiting diverse profiles covering important countries or geographical areas, institutions, interest groups and scientific fields for wheat research. The Wheat Initiative board was instrumental to identify missing profiles. An important success factor has been to include in this group all the key players of international wheat research. This initial group have been complemented by new members along all these years. Being inclusive by nature, the EWG accepted researchers and developers willing to contribute to the project. They meet once a year in a face-to-face meeting and regularly using videoconferences in between. The current EWG members are from the following organizations: l’Unité de Recherche Génomique Info (URGI) at l’Institut national de la recherche agronomique (INRA) (France), University of Western Australia (Australia), National Institute of Agricultural Botany (NIAB) (UK), the U.S. Department of Agriculture, Agricultural Research Service (USDA-ARS) (USA), the International Maize and Wheat Improvement Center CIMMYT (Mexico), the European Bioinformatics Institute (EMBL-EBI) (UK), GARNet (UK), Agriculture and Agri-Food Canada (Canada), Oregon State University (USA), Earlham Institute (UK), University of California, Davis (USA), Howard Hughes Medical Institute (HHMI) (USA), James Hutton Institute (UK), MIPS (Germany), University of Saskatchewan (Canada), Rothamsted Research (UK), International Wheat Yield Partnership (USA), and Royal Botanic Gardens, Kew (UK).

*Seeking help from other communities*. Achieving data interoperability is a difficult task because of data and tool heterogeneity, but also because of social and scientific challenges. To help, the Wheat Data Interoperability Working Group (WDI-WG) was created as one of the Research Data Alliance working groups, under the umbrella of the WheatIS Expert Working Group. This group was built from scientists taken from diverse fields such as data sciences, web semantics, genomics, phenomics and genetics. Some members belong to the WheatIS EWG, other have a more fundamental or transversal interest. Interestingly, some of them come from communities of other species such as rice. They participate to the work to help defining guidelines for their own community, taking advantage of the diversity of skills brought by the group, but also help us to be more generic in the proposed guidelines. This was a good insurance for the long-term sustainability of our proposals. Moreover, that also demonstrated how our approach can be generalized to other species, so that we found that our experience is valuable outside our community.

*The starting action*. Our first common action was to start working on surveys, interrogating the wheat research community on the usage of data standards in the wheat research community through a series of questions sent out to researchers and stakeholders in wheat science. The questions and answers were reported to the community ( Subirats *et al.*, 2015). Our successful process leading to the proposed guidelines was described in a community paper ( Dzale Yeumo *et al.*, 2017).

### Funding for WheatIS and WheatIS EWG

The Wheat Initiative serves as an umbrella organization for eleven EWGs and provides a loose connection between the EWGs to interact, however, it provides very limited funding for the EWGs to meet and organize workshops. For example, in 2018, approximately 9,000 euros were provided by the Wheat Initiative to partially subsidize attendee costs for an annual meeting that took place as a side meeting at the Plant and Animal Genome Conference in San Diego, CA and two workshops in Europe. To this date, no salary is provided to WheatIS EWG members or the members of their research groups to create or contribute to wheatis.org.

This meager funding from the Wheat Initiative means that many people that are involved in the WheatIS EWG activities, such as curating data, building indexed data sets, configuring and maintaining servers, are doing these tasks on a volunteer basis in addition to their regular daily tasks. Fortunately, both computational and experimental research groups that are part of the WheatIS community recognize the primary importance of data availability, access, and sharing through wheatis.org, and because it is beneficial to the larger scientific community, they consider their service a crucial part of their scientific responsibility. This somewhat guarantees a relative long-term sustainability of the initiative.

### A successful result: Wheat Information System (wheatis.org)

The most significant accomplishment of the WheatIS EWG is the creation of a central hub, called WheatIS that provides a publicly available single-entry point. The WheatIS core server have access to resources at the globally-distributed nodes and enables data query and extraction through the web portal, unifying data discovery for the wheat research community.

Specifically, the WheatIS portal was created to: 1) provide access to a data file repository storing files with their associated metadata; 2) allow queries to find data available in the WheatIS core and its nodes using keywords through a google-like search engine; 3) Data standards recommendations ( Dzale Yeumo *et al.*, 2017); and 4) catalog several dedicated integrative databases that manage data types such as genomic, genetic, phenotypic, and functional genomic.

*Current WheatIS searchable nodes*. The following are the current organizations that manage a WheatIS server node: 1) the International Maize and Wheat Improvement Center CIMMYT (Mexico), 2) the European Bioinformatics Institute (EMBL-EBI) (UK), 3) the GrainGenes database (USA), 4) the Gramene database (USA), 5) the Triticeae Toolbox database (USA), 6) Transplant-IPGPAS (Poland), 7) l’Unité de Recherche Génomique Info (URGI) (France), and 8) wheatgenome.info at the University of Western Australia (Australia). Among them, the URGI node is the main server that queries other servers. Note that the actual contributor list provided previously is larger than the number of nodes, because some groups contribute their data through already existing nodes located at other organizations.

*Rules of how to become a part of the WheatIS community*. The WheatIS community is always expanding, adding new data sets and nodes from groups that never contributed data to wheatis.org. WheatIS contributing members provides know-how and support to those who would like to create and maintain their own WheatIS nodes at their locations or contribute data to WheatIS, a simple request to wheatis-contact@wheatis.org will provide help and support.

### Outreach

Good communication is crucial for the success of such an endeavor. In addition to the website, we set up a Twitter account and use a mailing list to inform on our activities. Regularly, the Wheat Initiative organized meetings of its EWGs. These are useful opportunity to show our progress, to discuss the needs of the wheat research community, and to demonstrate the usefulness of our contribution. We were regularly invited to international conference where we presented our goals and the results. All these events contributed largely to make our initiative known by a number of scientists.

But, more interestingly, beside these quite obvious actions, an important part of our strategy was to organize training in different circles. At these occasions, we presented our tools to make them adopted by more and more people, but we also got feedback on their usage and the needs that help us to improve our work. In particular we organized joint meeting with other EWG to better answer needs from some scientific communities. Hence the “phenomics” EWG under the Wheat Initiative benefited a lot from such interactions.

### What made WheatIS successful? Formula for success for other communities

Our primary goal for the WheatIS Expert Working Group is to create a single portal that can query indexed data sets distributed worldwide, extract information, and provide access to these data sets to the wheat research community. The task of creating a technical framework for a single portal with access to multiple nodes is now accomplished. The size and types of data sets accessible at WheatIS are growing daily with more nodes being added. In all apparent measures, WheatIS is successful in building up a highly collaborative community of wheat research groups and creating a valuable product that is useful in connecting heterogenous data sets. When other scientific communities learned about our success such as rice ( Scheben *et al.*, 2019), grapevine ( Adam-Blondon *et al.*, 2016), AgBioData ( Harper *et al.*, 2018), we were asked how we accomplished this challenging task, i.e., what our formula was for success. Consequently, some of our approaches have been followed by these groups ( Adam-Blondon *et al.*, 2016). In this section, we provide our perspective as a way to guide other current and future research communities.

*Keeping data distributed*. Keeping data in place of their existing repositories, working on improving their visibility, but also involving people who managed them was a strategy we chose since the beginning. Even if technically more difficult, we thought that it is a key decision that helped us to build a community of data managers. By this we acknowledge the contribution of each contributor to the system, offering them the visibility they need for their own sustainability. Keeping a consistent group of motivated people who can obtain rewards from their efforts when they share their data is essential. In addition, it helps them to obtain funding from their own institutions or countries. This win-win strategy was a key determinant for the long-term success of our group. The social aspect of such a project is needed to be carefully considered and certainly not neglected when facing technological challenges. Here we preferred perhaps to make the challenge technically more difficult by emphasizing and prioritizing the social dynamics and group cohesion.

*Identifying a burning need (a.k.a. “an overarching and shared vision”)*. The primary starting point to create a community group is to identify a burning need around which the community should be formed. Only such a critical need will encourage researchers to go out of their institutional bubbles to navigate through complicated policies and procedures across national lines and devote their times voluntarily. A group of people can only function as a community as long as a burning need exists. If a need loses its importance and a new burning need is not identified, then the buy-in from scientists weakens and the community falters. If on the other hand, a new need is identified, a community can be transformed, and even evolved with the injection of new people energizing the community, even in some cases replacing some of the “old guard” in the process. In the case of WheatIS, the remarkable need to search, reach, and extract wheat data sets that are generated across the globe was a given among wheat scientists, and still energizes the community as more data sets are being generated with continually cheapening experimental technologies and computational power.

*Leadership principles*. One of the important features that define a research community is its leadership rational. Cooperation and trust for mutual benefit are of paramount importance. Such an endeavor requires to devote for the needs of the community, so that the community members follow these examples and respond positively. Another important point to emphasize here is that experience and skill sets for managing people and projects are essential. Although it is important for a research community to have leaders who are accomplished scientists, it does not necessarily mean that all accomplished scientists can lead the community to the next level diplomatically and successfully. Natural leadership should take precedence over exceptional publication record; the lack of publications in glamorous journals should not preclude someone to become a leader.

*Creating a supra-institutional umbrella group with broad appeal*. When establishing a research community, it is also important that the umbrella group should preferably not be led by a single institution, but a wide range of institutions, hopefully international, to create a broad appeal to attract scientists from different institutions. Institutions with big names can provide a great impetus at the beginning, especially with scientists and institutions that are already collaborating with the researchers in those institutions, but then with a single institution, there is a greater chance for the initial momentum to stall with time, and it is a better move to rely on multiple institutions, in a sense to diversify the risk of relying on a single institution. Also, instead of starting with well-known institutions, an alternative is to create an organization above (i.e., “supra-) the partnering organizations, so that partnering organizations feel that they are not being led by a well-known institute, but they are partners on equal terms with each institution under the umbrella group. A feeling of equality will create a greater buy-in from organizations and scientists. In the case of WheatIS EWG, the formation of the Wheat Initiative by G20 ministers of agriculture instantly created such a supra-national umbrella organization. Two crucial aspects presented an opportunity to start such an international organization for wheat: 1) wheat is among the top three crops in the world and 2) it has been produced by a large number of nations in all the continents except Antarctica ( Dubcovsky & Dvorak 2007).

Not every supranational organization need to be close-knit and built top-down. For example, the *Arabidopsis* community went through a stage where the National Science Foundation (NSF) in the U.S. steadily reduced the funding for the centralized *Arabidopsis* database TAIR ( Reiser *et al.*, 2017), forcing the community to seek funding from the funding agencies in different nations to keep the database. In this bottom-up case, the International Arabidopsis Informatics Consortium was formed ( International Arabidopsis Informatics Consortium 2010; International Arabidopsis Informatics Consortium, 2019) and provided a venue for scientists and national funding agencies to exchange ideas to support *Arabidopsis* informatics structure and reached consensus among organizations to maintain and improve on the community’s informatics structure. It is also important to mention that some members of the WheatIS EWG are also members of the maize, *Brassica* and rice communities and their contributions played a significant role in WheatIS’s success and in turn their experience in the WheatIS initiative are making an impact in their communities.

*Broad range of deep, dedicated scientific expertise*. The Wheat Information System needed a wide range of expertise to make wheatis.org a reality. It needed technical expertise to build and maintain a strong computational infrastructure and create data formats to make data sets readable; scientific expertise to understand different types of wheat data sets (including genetic, genomic, phenotypic, and metabolic); outreach capability to help build relationships to add new nodes with new data sets; and leaders who not only motivate and manage personnel, but also work with the Wheat Initiative and the broader wheat community to promote and support WheatIS. The need for dedicated and competent personnel with complimentary and overlapping expertise was crucial. For WheatIS, or for any scientific community for that matter, the critical question is the type of the expertise needed and how much time the experts can devote to a fledgling community.

## Conclusions and future work

### Wheat Information System as the focal point of Wheat Initiative

When the WheatIS EWG was formed to create a single portal to make wheat data sets findable, accessible, and shareable ( Wilkinson *et al.*, 2016a), the initial focus was primarily on the data sets. However, sharing data sets also lead to strengthening wheat communities as well, which happened for WheatIS working under the Wheat Initiative. Through WheatIS and through sharing data sets, WheatIS has evolved into a fledgling nexus for the other EWGs, a few in the beginning, and more later, to contribute to a single portal where any data points generated would be made accessible. Sharing data does not only require creating data sets in a certain data format and placing them in a certain data directory on a server, but it also requires communication and planning between research groups and between Expert Working Groups. Through this communication network, WheatIS is helping the Wheat Initiative to become a more cohesive group and facilitates future collaborations. These types of collaborations will have a larger impact beyond the Wheat Initiative, first through the wheat research groups that are not part of the Wheat Initiative, and later other plant researchers and researchers working with other species.

### Developing common gene nomenclature standards

The collaboration across EWGs to develop common data standards is an ongoing effort between and within Expert Working Groups. Following the workshops previously organized in 2017 in Tulln, Austria and Berlin, Germany, and recently in 2019 in the Wheat Initiative Research Committee meeting at the First International Wheat Congress in Saskatoon, Canada, a decision was taken to broaden the participation by including people from other EWGs, and another workshop is in the planning stages to create guidelines for gene naming for genetics and genomics data.

### WheatIS 2.0

Although the current graphical user interface for WheatIS is functional, it needs improvement in several areas. There are ongoing efforts to create a more user-friendly interface to improve user experience. It is not straightforward for users to identify where and how to start their search intuitively, and we plan to provide more information and support links for users. In the new interface, which we colloquially name WheatIS 2.0, we plan not only to work with the cosmetic issues, but also functional issues such as providing a more advanced and easy-to-use search capabilities. Currently some advanced search features are offered to users, but only *after* a search term is entered and *when* search results are shown. We plan to incorporate an Advanced Search feature without the need to enter a search term first to show the range of data types. We also need to improve our semantic search capabilities, considering the recent advances in the field. The WheatIS 2.0 will be shaped in these and other specific areas that were identified through personal discussions in the EWG meetings and the feedback we received from actual users.

## Data availability

### Underlying data

No data are associated with this article
