# Supplementary material for: Building a successful international research community through data sharing: The case of the Wheat Information System (WheatIS)
Source: F1000Res. 2020 Jun 5;9:536. [Version 1] doi: 10.12688/f1000research.23525.1 (PMC7953914; doi:10.12688/f1000research.23525.1)
Supplement: Peer review history

# Peer review: Reviewer response for version 1

**DOI:** 10.5256/f1000research.25960.r75739

**Published:** 2021-03-11

**Authors:** Nevin Gerek Ince

**Affiliations:** 1 AVEVA, Houston, TX, USA

Sen *et.al. *described their experience and learning in order to establish an international research community to build a data infrastructure, standards and a single portal for wheat data sets around the world. I think the manuscript is clearly written and explains their endeavours and challenges to build wheatis.org website. Their experience provides a guidance for research communities how to create global tools and resources. I should emphasize that their experience is not only specific to the Wheat Initiative with a broad range of contributing organizations. Their challenges are also valid for other research communities.

 I wonder if they are planning to provide some data analytics or AI type of data prediction in WheatIS in the future.

Is the topic of the opinion article discussed accurately in the context of the current literature?

Yes

Are arguments sufficiently supported by evidence from the published literature?

Yes

Are all factual statements correct and adequately supported by citations?

Yes

Are the conclusions drawn balanced and justified on the basis of the presented arguments?

Yes

Reviewer Expertise:

NA

I confirm that I have read this submission and believe that I have an appropriate level of expertise to confirm that it is of an acceptable scientific standard.

# Peer review: Reviewer response for version 1

**DOI:** 10.5256/f1000research.25960.r79260

**Published:** 2021-02-15

**Authors:** Stavros Makrodimitris

**Affiliations:** 1 Delft Bioinformatics Lab, Delft University of Technology, Delft, The Netherlands

The manuscript by Sen *et al. *describes a community-wide effort to create a large information resource about the wheat genome. The authors share their experience to be used as a potential guide for similar endeavors in the future. The manuscript is concise and clearly written.

 I have a couple of suggestions to the authors from the perspective of bioinformatics:

1. Datasets from such large databases often contain large ‘batch effects’, i.e. technical confounders such as differences in experimental protocols, sequencing depth etc. Such batch effects make joint analysis of different datasets more challenging.  Are there any efforts to provide batch-corrected data or are only the ‘raw’ data available?
2. The authors mention plans to improve the user interface, but as I expect the data in such a big database to continue growing, sooner or later there might be a need for some kind of programmatic access through, for example, an API or an R package. Does such a tool exist? If not, could the authors mention what their plans are in that direction?

Is the topic of the opinion article discussed accurately in the context of the current literature?

Yes

Are arguments sufficiently supported by evidence from the published literature?

Yes

Are all factual statements correct and adequately supported by citations?

Yes

Are the conclusions drawn balanced and justified on the basis of the presented arguments?

Yes

Reviewer Expertise:

bioinformatics, plant gene function prediction

I confirm that I have read this submission and believe that I have an appropriate level of expertise to confirm that it is of an acceptable scientific standard.

# Peer review: Reviewer response for version 1

**DOI:** 10.5256/f1000research.25960.r64353

**Published:** 2020-06-26

**Authors:** Runxuan Zhang

**Affiliations:** 1 Information and Computational Sciences, James Hutton Institute, Invergowrie, UK

The manuscript described the process and successful experiences of forming an international research community, namely the international Wheat Information System (WheatIS) Expert working group. The focus of this article is on the higher level identifying the key factors to build a sustainable and successful community, such as establishing an urgent and shared vision, drawing in broad areas of deep and dedicated expertise, setting up an umbrella group with broad appeal and equal footing for partners, etc. Some technical aspects were also touched on such as keeping the data distributed and setting up a core with access to globally distributed nodes. 

 Although technical issues are not the focus of the paper, here are a few comments regarding to wheatIS.org:

1. On its official webpage, a few tabs on the wheatIS.org, such as "submit data" and "tools" are still under construction and not available for use.
2. One of the aims of the EWG is to allow people to share their data. What is the procedures in place to check the quality of the data? How does the system prevent false info from getting into the system?
3. Currently there is very limited funding available for this initiative. Most of the work is done on a volunteer basis. For the long term sustainability, the values created for end-user of the wheatis.org and impact it could make should be considered. A route for funding should be established through charity, governmental funding or industry to fund the initiative directly considering the full economic cost. 

Is the topic of the opinion article discussed accurately in the context of the current literature?

Yes

Are arguments sufficiently supported by evidence from the published literature?

Yes

Are all factual statements correct and adequately supported by citations?

Yes

Are the conclusions drawn balanced and justified on the basis of the presented arguments?

Yes

Reviewer Expertise:

high throughput data analysis in plant species, transcriptomics and proteomics

I confirm that I have read this submission and believe that I have an appropriate level of expertise to confirm that it is of an acceptable scientific standard, however I have significant reservations, as outlined above.

# Response

**Published:** 2020-09-01

**Authors:** Hadi Quesneville

**Affiliations:** INRAE Versailles, France

We thank Runxuan Zhan from the James Hutton Institute (UK) for this review. Below are the replies to its comments.

**Reviewer comment #1**: On its official webpage, a few tabs on the wheatIS.org, such as "submit data" and "tools" are still under construction and not available for use.

**Response**: We thank the reviewer for his thoroughness. The header links are now fully functional.

**Reviewer comment #2**: One of the aims of the EWG is to allow people to share their data. What are the procedures in place to check the quality of the data? How does the system prevent false info from getting into the system?

**Response**: We agree with the reviewer that data quality is one of the most crucial aspect of data availability and stewardship. At the lowest level of quality check, when a new dataset is indexed at one of the nodes, the WheatIS system automatically checks its formatting, and if the dataset is not formatted correctly, it is not displayed through the web interface, and flagged for further quality check. 

 Although correct formatting and accurate display of the datasets are important, the most important criterion for indexing a dataset at WheatIS is primarily its scientific value for the WheatIS users. The process of selecting and indexing datasets at the WheatIS nodes is crucial; every dataset available through the WheatIS framework needs to be high-quality. Therefore, all indexed datasets at WheatIS are required to be either peer-reviewed, manually curated by professional curators, or both. Most datasets at WheatIS are the products of peer-reviewed scientific research with associated peer-reviewed publications. Some others, such as Wheat Gene Catalogue, are being curated for decades and approved by a committee of wheat genetics experts. 

**Reviewer comment #3**: Currently there is very limited funding available for this initiative. Most of the work is done on a volunteer basis. For the long-term sustainability, the values created for end-user of the wheatis.org and impact it could make should be considered. A route for funding should be established through charity, governmental funding or industry to fund the initiative directly considering the full economic cost.

**Response**: We thank the reviewer for bringing this issue to our attention, as well as, the attention of the funding agencies. In addition to our ongoing volunteer efforts, we continuously look for new funding resources for WheatIS.  
